# Supplementary material for: Surface-Pore-Modified N-Doped Amorphous Carbon Nanospheres Tailored with Toluene as Anode Materials for Lithium-Ion Batteries
Source: Nanomaterials (Basel). 2024 Apr 28;14(9):772. doi: 10.3390/nano14090772 (PMC11085129; doi:10.3390/nano14090772)
Supplement: Supplementary file 1 [file nanomaterials-14-00772-s001.zip › nanomaterials-2929386-supplementary.pdf]

Supporting information

Surface-Pore-Modified N-Doped Amorphous Carbon Nanospheres Tailored with Toluene as Anode Materials for Lithium-Ion Batteries

Shiran Shan [1], Chunze Yuan [1,2,*], Guangsu Tan [1], Chao Xu [1], Lin Li [1,2], Guoqi Li [1], Jihao Zhang [1] and Tsu-Chien Weng [1,2,*]

1 School of Physical Science and Technology, ShanghaiTech University, Shanghai 201210, China; shanshr@shanghaitech.edu.cn (S.S.); tangs@shanghaitech.edu.cn (G.T.); xuchao1@shanghaitech.edu.cn (C.X.); lilin1@shanghaitech.edu.cn (L.L.); ligq1@shanghaitech.edu.cn (G.L.); zhangjh5@shanghaitech.edu.cn (J.Z.)
2 Center for Transformative Science, ShanghaiTech University, Shanghai 201210, China
* Correspondence: yuanchz@shanghaitech.edu.cn (C.Y.); wengzq@shanghaitech.edu.cn (T.-C.W.)

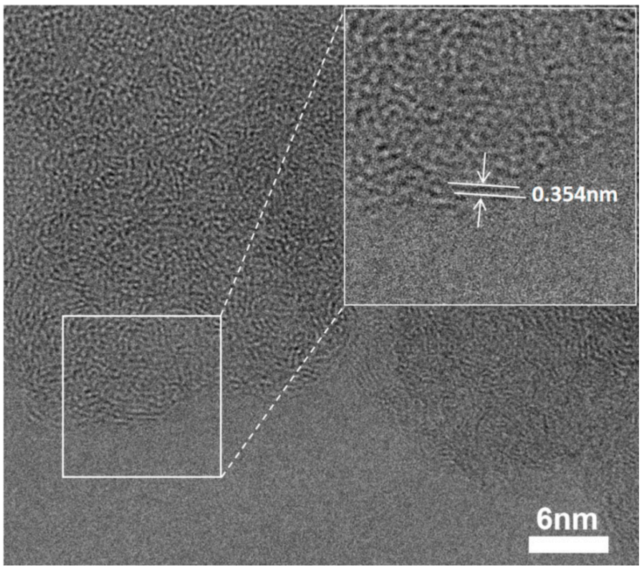

Figure S1. HR-TEM images of ACNs-100.

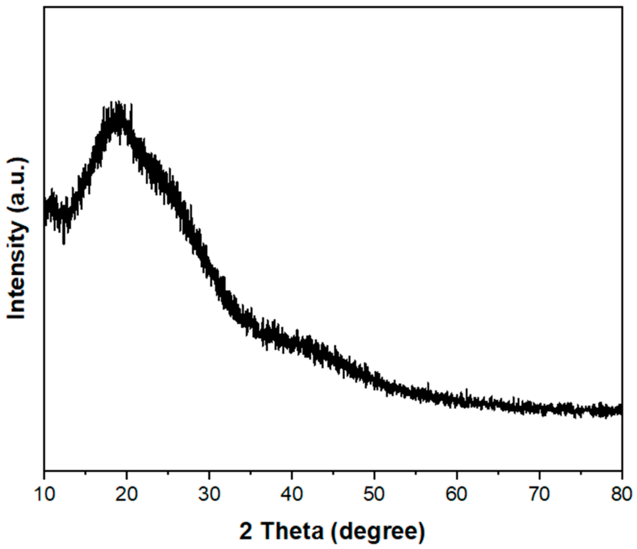

Figure S2. XRD pattern of resin nanospheres.

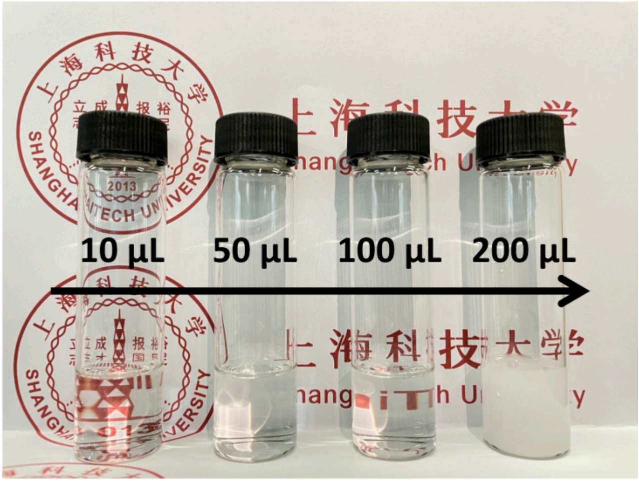

Figure S3. Images of the resulting F-127/toluene/TAPB nanoemulsions with the addition of 10 μL, 50 μL, 100 μL, and 200 μL toluene.

Table S1. Specific surface areas of ACNs.

Sample	ACNs-10	ACNs-50	ACNs-100	ACNs-200
S_{BET} (m^2 g^{-1})	557.9	610.7	618.8	594.5
V_{MICRO} (m^3 g^{-1})	0.212	0.198	0.191	0.177
S_{MICRO} (m^2 g^{-1})	526.8	482.9	462.9	426.4
S_{EX} (m^2 g^{-1})	31.1	127.8	155.9	168.1

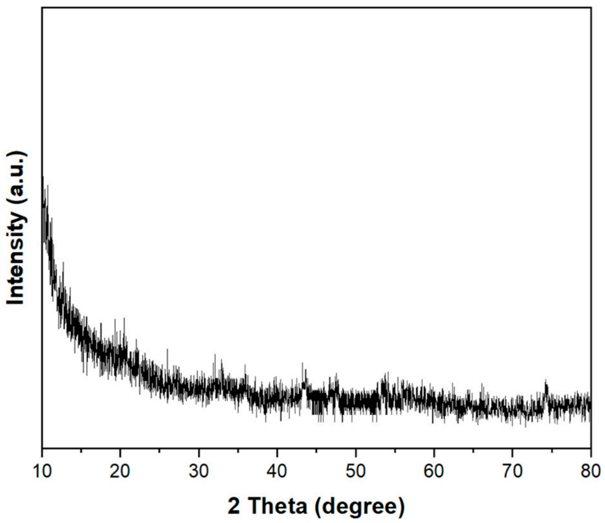

Figure S4. XRD pattern of ACNs-100 after cycling.
